# Green Nut Oil or DHA Supplementation Restored Decreased Distribution Levels of DHA Containing Phosphatidylcholines in the Brain of a Mouse Model of Dementia

**DOI:** 10.3390/metabo10040153

**Published:** 2020-04-16

**Authors:** Ariful Islam, Emiko Takeyama, Md. Al Mamun, Tomohito Sato, Makoto Horikawa, Yutaka Takahashi, Kenji Kikushima, Mitsutoshi Setou

**Affiliations:** 1 Department of Cellular and Molecular Anatomy, Hamamatsu University School of Medicine, Handayama, Higashi-ku, Hamamatsu, Shizuoka 431-3192, Japan; ariful222222@gmail.com (A.I.); amamun5245@gmail.com (M.A.M.); titon0620@gmail.com (T.S.); makotoh@hama-med.ac.jp (M.H.); yutaka.ironman@gmail.com (Y.T.); largo.kenji@gmail.com (K.K.); 2 Department of Food Science and Nutrition, Graduate School of Human Life Sciences, Showa Women’s University, Taishido, Setagaya-ku, Tokyo 154-8533, Japan; takeyama@swu.ac.jp; 3 Institute of Women’s Health Sciences, Showa Women’s University, Taishido, Setagaya-ku, Tokyo 154-8533, Japan; 4 International Mass Imaging Center, Hamamatsu University School of Medicine, Handayama, Higashi-ku, Hamamatsu, Shizuoka 431-3192, Japan; 5 Department of Systems Molecular Anatomy, Institute for Medical Photonics Research, Preeminent Medical Photonics Education & Research Center, Handayama, Higashi-ku, Hamamatsu, Shizuoka 431-3192, Japan

**Keywords:** dementia, green nut oil, DHA, SAMP8 mice, DHA-PCs, DESI-MSI

## Abstract

Dementia is a major public health concern nowadays. Reduced levels of brain docosahexaenoic acid (DHA) and DHA-phosphatidylcholines (DHA-PCs) in dementia patients were reported previously. Recently, we have reported that supplementation of green nut oil (GNO) or DHA improves memory function and distribution levels of brain DHA in senescence accelerated mice P8 (SAMP8). GNO is extracted from *Plukenetia volubilis* seeds, and SAMP8 is a well-known model mouse of dementia. In this current study, we examined the results of GNO or DHA supplementation in the distribution levels of brain DHA-PCs in same model mouse of dementia using desorption electrospray ionization (DESI) mass spectrometry imaging (MSI). We observed significantly decreased distribution of brain DHA-PCs, PC (16:0_22:6), and PC (18:0_22:6) in SAMP8 mice compared to wild type mice, and GNO or DHA treatment restored the decreased distribution levels of PC (16:0_22:6) and PC (18:0_22:6) in the brain of SAMP8 mice. These results indicate that GNO or DHA supplementation can ameliorate the decreased distribution of brain DHA-PCs in dementia, and could be potentially used for the prevention and treatment of dementia.

## 1. Introduction

Dementia is one of the major global health problems mainly in elderly people in which there is deterioration in memory, thinking, behavior, and the ability to perform daily activities [1]. As the world’s population is aging rapidly, dementia has become a concern throughout the world, and it places substantial burden on patients having dementia [2]. In 2018, the total global cost of dementia care was estimated at US$ 0.948 trillion with 15.94% annual growth rate [3]. Now, nearly 0.05 billion people worldwide are suffering from dementia, and it is projected to reach about 0.135 billion by 2050 [1]. Despite the high prevalence and economic burden of dementia, disease-modifying therapies are yet to be developed.

Among all omega-3 fatty acids that have been detected in animal brain, the most abundant omega-3 fatty acid is docosahexaenoic acid (DHA) [4]. It incorporates into phosphatidylcholines (PCs), major lipid components of the brain [5,6]. Brain DHA and DHA-PCs play a significant role to regulate protein–protein interaction, and required for optimal neuronal function [7]. They also regulate membrane fluidity by controlling the function of synaptic membrane-associated proteins [7]. Several studies have reported that the patients having dementia have decreased brain DHA and DHA-PCs [8,9]. Although DHA supplementation improves the accumulation of brain DHA in dementia mouse model, the effects of the supplementation of DHA on the distribution of brain DHA-PCs in dementia are not known yet [10]. Therefore, we conducted this study to evaluate changes in the distribution levels of brain DHA-PCs in senescence accelerated mice P8 (SAMP8) after DHA supplementation. SAMP8 mouse is a model mouse of dementia [11]. It shows several characteristics of dementia, such as progressive deficits in learning, decline in memory, dysfunction of blood–brain barrier (BBB), degeneration of the brain stem, oxidative stress mediated damage, apoptotic death of neuronal cell, alternation of amyloid-β (Aβ), and hyperphosphorylation of tau [11,12].

Currently, the increased demand for fish oil DHA due to its tremendous health benefits has created a notable effects on marine population, and it is believed that large scale fishing from sea will become unsustainable in near future [13]. Therefore, alternative and more sustainable source for DHA is required in near future. Oil produced from *Plukenetia volubilis* seeds is known as green nut oil (GNO). It contains several fatty acids including about 50% alpha-linolenic acid [14]. It acts as a precursor for DHA biosynthesis in mammals [13]. Therefore, GNO has the potentiality to become an alternative source of DHA. Moreover, GNO is cheaper than DHA. As like as DHA, GNO also improves memory function and brain DHA levels in SAMP8 mice [10]. In this study, we have also examined the effect of GNO supplementation in the distribution levels of brain DHA-PCs in SAMP8 mice.

Mass spectrometry imaging (MSI) is widely used for the detection and visualization of biomolecules in the samples [15,16,17,18]. In last decade, desorption electrospray ionization MSI (DESI-MSI), a novel MSI tool was developed. DESI-MSI does not require matrix, and it permits the identification and visualization of molecules such as drugs, metabolites, lipids, and proteins under ambient condition [10,19,20]. Compared to other conventional techniques of MSI, DESI-MSI is minimally destructive and require minimal sample preparation [21,22]. Our group already analyzed several samples using this novel technique [10,16,19,20]. In this study, we applied this technique to reveal the effects of GNO or DHA supplementation in the distribution levels of brain DHA-PCs in SAMP8 mice.

## 2. Results

### 2.1. Detection of Peaks Corresponded to DHA-PCs from DESI-MSI Spectra

**Figure 1:**
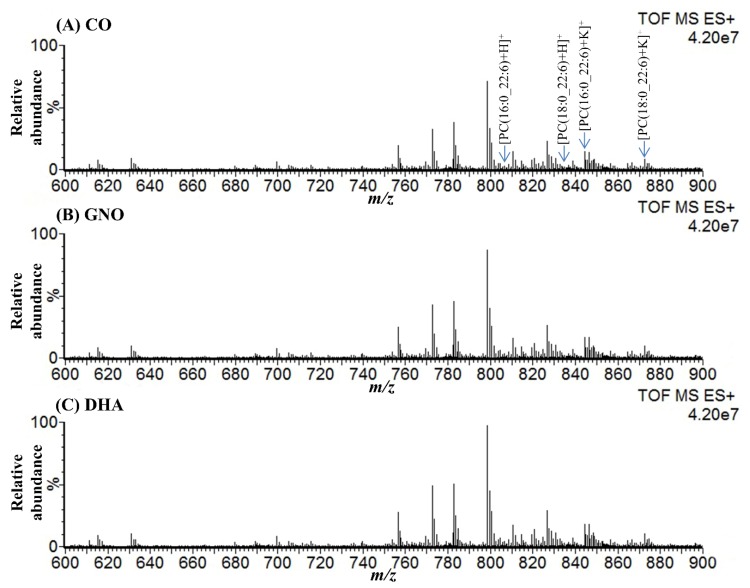
Representative DESI-MSI mass spectra acquired in positive ionization mode from the sagittal brain sections of SAMP8 mice. CO: corn oil treated SAMP8 mice; GNO: green nut oil treated SAMP8 mice; DHA: docosahexaenoic acid (C22:6) treated SAMP8 mice; PC: phosphatidylcholine, and *m/z*: mass to charge ratio.

DESI-MSI mass spectra were acquired from the sagittal brain sections of 14-week-old wild type (WT) mice (untreated), 14-week-old SAMP8 mice (untreated), and 28-week-old SAMP8 mice treated with CO, GNO, and DHA in positive ion mode over 500−1000 *m/z* (mass/charge ratio) range to explore the changes of DHA-PCs distribution after GNO or DHA supplementation. Figure 1 and Figure S1 show the representative mass spectra obtained from WT and SAMP8 mice brains. In total, four peaks corresponded to the molecular ions of DHA-PCs were selected from the mass spectra acquired from all mice groups with *m/z* 806.6, 834.6, 844.5, and 872.6 (Figure 1 and Figure S1).

### 2.2. Molecular Identification of DHA-PCs by Tandem Mass Spectrometry

**Figure 2:**
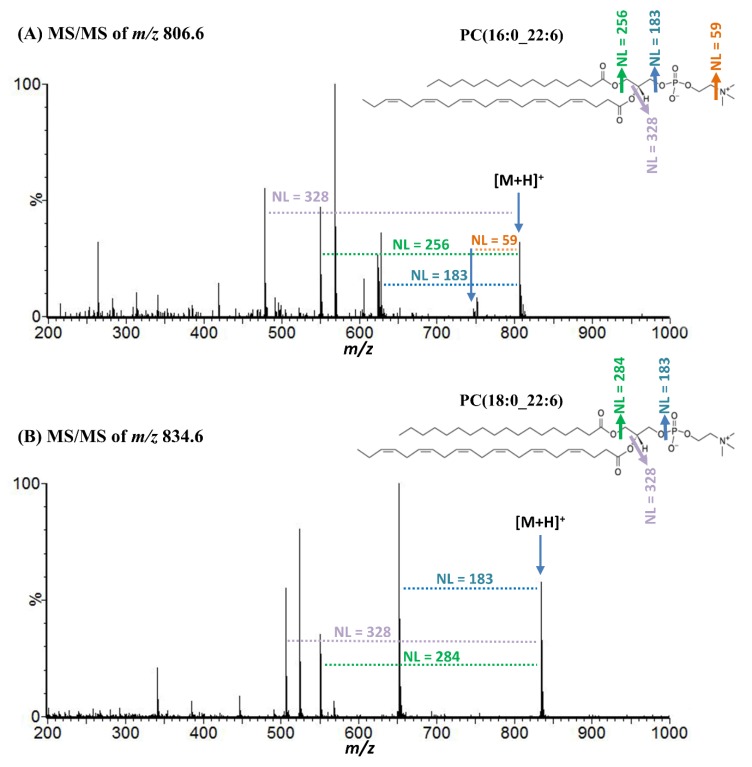
Representative LC-ESI-MS/MS spectra of *m/z* 806.6 (**A**) and 834.6 (**B**) in positive ionization mode. NL; neutral loss.

To assign molecules against *m/z* 806.6, 834.6, 844.5, and 872.6, liquid chromatography (LC) electrospray ionization (ESI) tandem mass spectrometry (MS/MS) analysis was performed (Figure 2). Neutral losses of (NL) 59, 183, 256, 284, and 328 Da from parent ions were corresponded to trimethylamine, phosphocholine, palmitic acid, stearic acid, and DHA, respectively (Figure 2) [23,24,25].

NL of 38 u from precursor ions with *m/z* 844.5 and 872.6 indicates potassium adduct forms (Table 1) [23]. Several other product ions were detected in the LC-MS/MS spectra of *m/z* 806.6, 834.6, 844.5, and 872.6, which were also reported previously (Table 1) [23,24,25].

### 2.3. Distribution Levels of DHA-PCs in the Brain of 14-Week-Old WT and SAMP8 Mice

**Figure 3:**
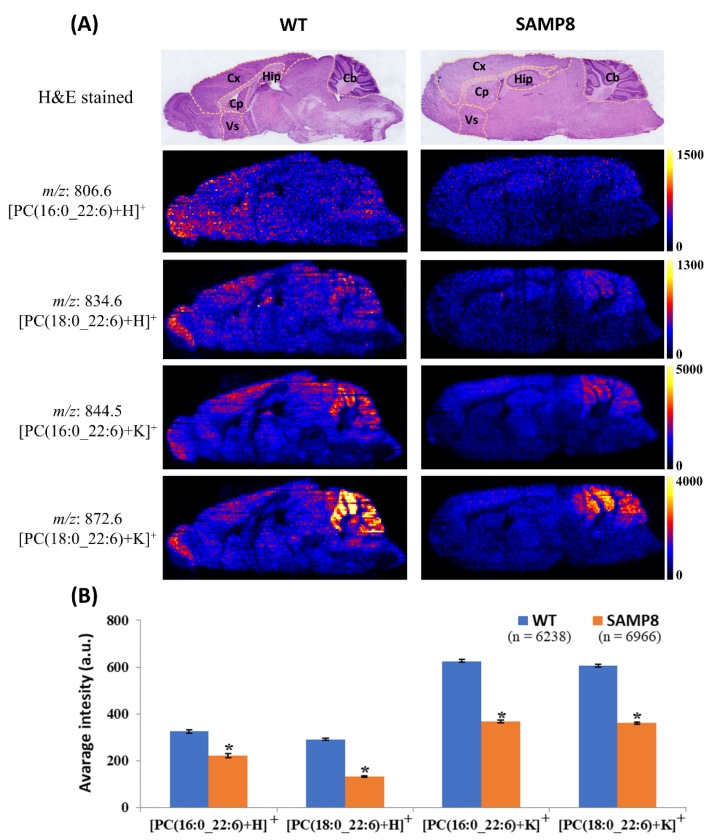
Differences in the distribution levels of brain DHA-PCs between SAMP8 mice and WT mice. (**A**) Representative ion images of DHA-PCs in the brain of 14-week-old SAMP8 mice and 14-week-old WT mice. Here Cb, Cx, Hip, Cp, and Vs indicate cerebellum, cerebral cortex, hippocampus, caudate putamen, and ventral striatum, respectively. (**B**) Average intensity of DHA-PCs in whole sagittal section of 14-week-old WT and SAMP8 mice. All values are represented as mean ± SEM. Here ‘*’ indicates *p* < 0.05 (two-tailed *t*-test), ‘*n*’ indicates number of pixels, and ‘a.u.’ indicates arbitrary unit.

In our previous study, we found decreased levels of brain DHA-PCs in dementia patients [8]. In this study, at first, we observed the differences in the distribution levels of brain DHA-PCs of 14-week-old SAMP8 mice compared to WT mice. Applying DESI-MSI, we found significantly decreased distribution levels of PC (16:0_22:6) and PC (18:0_22:6) in 14-week-old SAMP8 mice brain compared to WT mice brain (Figure 3 and Figure S2). Changes in the spatial distribution of brain DHA-PCs in mice were found in this study (Figure 3). Therefore, distribution of brain DHA-PCs in several regions, including hippocampus, which is associated with memory function were analyzed [26]. We found that the distribution of DHA-PCs largely decreased in the hippocampus, cerebellum, cerebral cortex, caudate putamen, and ventral striatum of SAMP8 mice brain compared to WT mice brain (Table S1). 

### 2.4. Accumulation of Brain DHA-PCs in GNO or DHA Treated SAMP8 Mice

**Figure 4:**
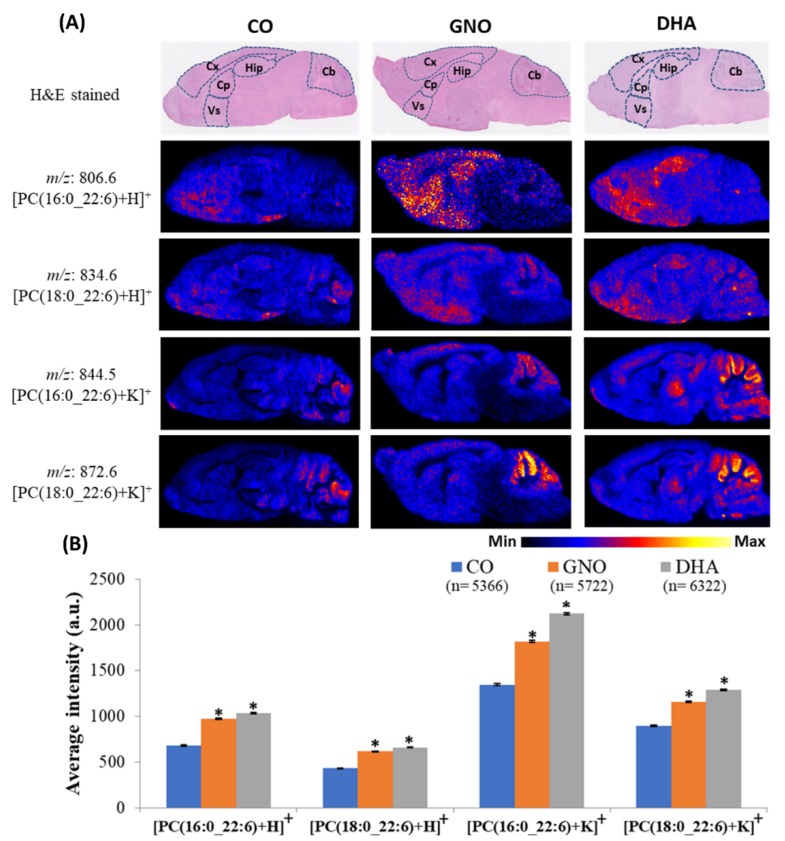
Effects of GNO or DHA treatment in the distribution levels of DHA-PCs in the brain of 28-week-old SAMP8 mice. (**A**) Representative ion images of DHA-PCs in the brain of CO, GNO, and DHA treated SAMP8 mice. Here Cb, Cx, Hip, Cp, and Vs indicate cerebellum, cerebral cortex, hippocampus, caudate putamen, and ventral striatum, respectively. (**B**) Average intensity of DHA-PCs in whole sagittal section of 28-week-old SAMP8 mice treated with CO, GNO and DHA. All values are represented as mean ± SEM. Here ‘a.u.’ indicates arbitrary unit, ‘*’ indicates *p* < 0.05 (one-way ANOVA with Tukey’s multiple comparison), and ‘*n*’ indicates number of pixels.

Recently, we have reported that fourteen weeks treatment with GNO or DHA improves brain DHA levels and memory function in SAMP8 mice [10]. Therefore, in this study, we analyzed the distribution levels of brain DHA-PCs in SAMP8 mice treated with GNO or DHA for 14 weeks (Figure 4 and Figure S3).

Compared to CO treated SAMP8 mice, GNO or DHA treatment significantly increased the accumulation of PC (16:0_22:6) and PC (18:0_22:6) in SAMP8 mice brain. Accumulation of DHA-PCs in several brain regions of SAMP8 mice was also analyzed in this study. We observed largely increased distribution of DHA-PCs in the hippocampus, cerebellum, cerebral cortex, caudate putamen, and ventral striatum of GNO and DHA treated SAMP8 mice brain compared to CO treated SAMP8 mice.

## 3. Discussion

The results of this study proposed that GNO or DHA treatment could restore the decreased distribution levels of brain DHA-PCs, PC (16:0_22:6) and PC (18:0_22:6) in SAMP8 mice, a model mouse of dementia. Additionally, increased accumulation of DHA-PCs were also observed in several brain regions of GNO or DHA treated SAMP8 mice. As far as we know that this is first report regarding the improvement of brain DHA-PCs distribution after GNO or DHA treatment in model mice of dementia. 

In a previous study, we found decreased levels of brain DHA-PCs in dementia patients which were associated with neuronal loss and synaptic disruption which occurs in dementia [8]. DHA-PCs are the structural components of mammalian cell, and well known for numerous brain functions. In brain, DHA-PCs control the functions of proteins associated with synaptic membrane by regulating protein–protein interactions and membrane fluidity [7,9]. They also paly major role to regulate genes expression associated with signal transmission, act as precursors for second messenger, reduce oxidative stress, and inhibit inflammation [9,27,28]. Several recent studies also demonstrated that DHA-PCs are important to maintain memory and cognitive function of brain [28,29,30]. In this study, we applied DESI-MSI, a recently developed MSI technique to examine the distribution levels of DHA-PCs in the brain of WT and SAMP8 mice. MSI allows us the identification and visualization of drugs, metabolites and biomolecules [15,19]. Peaks of interest corresponded to DHA-PCs were detected from DESI-MSI mass spectra acquired from the sagittal brain sections of WT and SAMP8 mice. Applying LC-ESI-MS/MS analysis, molecule corresponded to ions with *m/z* 806.6 and 844.5 was confirmed as PC (16:0_22:6), and molecule corresponded to ions with *m/z* 834.6 and 872.6 was confirmed as PC (18:0_22:6) based on their fragmentation patterns and previous reports [23,24,25]. 

SAMP8 mouse is well known as a mouse model of dementia [11,31]. Although decreased brain DHA-PCs are associated with dementia, DHA-PCs levels in the brain of SAMP8 mice has remained unexplored [8]. Therefore, we analyzed the distribution levels of brain DHA-PCs of 14-week-old WT mice and 14-week-old untreated SAMP8 mice. Significantly decreased distribution levels of PC (16:0_22:6) and PC (18:0_22:6) were observed in SAMP8 mice brain compared to that of WT mice brain. Decreased distribution of those DHA-PCs were also observed in several brain regions including hippocampus, cerebellum, cerebral cortex, caudate putamen, and ventral striatum. These data suggest that similar to dementia patients, SAMP8 mice also have decreased distribution levels of DHA-PCs in whole brain (sagittal section) as well as several brain regions.

In our recent study, we have reported that fourteen weeks supplementation of GNO or DHA improves memory function and DHA accumulation in the brain of SAMP8 mice [10]. Therefore, in this study, we treated SAMP8 mice by CO, GNO, and DHA for 14 weeks and their brains were analyzed using DESI-MSI. DESI-MSI revealed that GNO or DHA supplementation could improve PC(16:0_22:6) and PC(18:0_22:6) distribution levels in the brains of SAMP8 mice. Several researchers have reported decreased brain DHA-PCs in patients with dementia due to altered metabolism of lipids [8,9,32]. Supplementation of α-linolenic (major constituent of GNO) or DHA can be incorporated into DHA-lysoPC [7,13,14]. DHA-lysoPC and free DHA can cross the BBB by passive diffusion and major facilitator superfamily domain-containing protein 2A, respectively [33,34]. Free DHA can incorporate into PCs and DHA-lysoPC of brain can be transformed into DHA-PCs by the action of lysoPC acyltransferase, and can improve the distribution levels of brain DHA-PCs in mice having dementia [35]. Therefore, our results of this study and previous reports suggest that GNO or DHA treatment could ameliorate the decreased distribution levels of brain DHA-PCs and improve memory function in dementia [10].

Among all regions of brain, the hippocampus has a significant role in memory function and its damage is associated with memory deficits in dementia patients [26]. Cerebral cortex interacts with hippocampus, and its damage is another common phenomena of dementia [36,37]. It has significant role to controls motor function, prospective memory, personality, and behavioral responses [38,39]. Several other brain regions including cerebellum, caudate putamen and ventral striatum are also vulnerable to dementia [40,41]. The cerebellum has significant roles in motor learning, motor activity, cognition, goal-oriented behavior, and emotion [42,43]. In the cerebellum, Aβ mediated neuronal damage and cognitive decline in dementia was reported previously [40,44]. Additionally, damage of caudate putamen and ventral striatum in dementia are also associated with the course of disease progression [41]. Both of caudate putamen and ventral striatum interconnect with different territories of frontal lobe, and control cognitive function [45,46]. Moreover, according to previous reports, DHA-PCs supplementation could improve memory function of SAMP8 mice by suppressing Aβ generation, neuro-inflammation, and apoptotic death of neuronal cells [29]. In this study, increased distributions of DHA-PCs in hippocampus, cerebellum, cerebral cortex, caudate putamen, and ventral striatum of SAMP8 mice were noted after GNO or DHA supplementation. Therefore, this study suggests that the supplementation of GNO or DHA could ameliorate decreased distribution of brain DHA-PCs, which could improve memory function and inhibit the progression of dementia. Currently, the elevated demand of fish oil for DHA, due to its immense health benefits, is becoming detrimental to fish numbers and species [47]. All data of this study also suggest that GNO could be a more sustainable source of DHA compared to fish oil. However, further investigations are required to explore the role of elevated brain DHA-PCs following GNO or DHA supplementation on other pathological hallmarks of dementia.

## 4. Materials and Methods 

### 4.1. Animal

Thirteen-week-old C57BL/6JJmsSlc and SAMP8 male mice were purchased from SLC Inc. (Hamamatsu, Japan) and Sankyo Labo Service Corporation, Inc. (Tokyo, Japan), respectively. All mice were reared at 12 h of light/dark cycle. Tap water and food were provided ad libitum. CRF-1 pellets consisting diet (Charles River International Laboratories, Inc., Kanagawa, Japan) were provided during first week of the preparatory periods. Three WT mice and three SAMP8 mice were dissected after first week of rearing period. After collection, all brain samples were stored at −80 °C until DESI-MSI analysis. All remaining SAMP8 mice were separated into three groups, and treated with CO, GNO, and DHA for 14 weeks as described by Takeyama et al. [10]. After 14 weeks of treatment, brain sample of all mice were collected after dissection, and stored at −80 °C until DESI-MSI analysis. All experiments were performed according to the protocols of Animal Care and Use Committee of the Hamamatsu School of Medicine, and Showa Women’s University Animal Research Committee (ethical approval number: 17-04).

### 4.2. Chemicals and Reagents 

Tama Biochemical Co., Ltd. (Tokyo, Japan) provided fish oil DHA-46, and NPO Arcoiris Naturaleza (Matsudo, Chiba, Japan) provided GNO. From Oriental Yeast Co., Ltd. (Tokyo, Japan), corn oil was purchased. Ammonium acetate, LC/MS grade 2-propanol and methanol, HPLC grade acetonitrile, chloroform, formic acid, acetic acid, and ultrapure water were purchased from Wako Pure Chemical Industries (Osaka, Japan). From Sigma-Aldrich (St. Louis, MO, USA), sodium formate was purchased. 

### 4.3. Group Size of Samples Analyzed by DESI-MSI

For DESI-MSI analysis, group sizes of mice were as follows: WT, *n* = 3; SAMP8 (untreated), *n* = 3; DHA-treated, *n* = 5; GNO-treated, *n* = 5 and CO-treated, *n* = 5. Three WT and three SAMP8 (untreated) mice were separated into three sets for DESI-MSI experiments by taking one mouse from both groups randomly. All 15 SAMP8 mice treated by CO, GNO, and DHA were divided into five experimental sets for DESI-MSI analysis by taking one mouse from all three groups for each experiment.

### 4.4. Preparation of Tissue Samples for DESI-MSI

Mice brains were frozen quickly in powdered dry ice after harvesting and stored at −80 °C. The brain tissues were sectioned to a thickness of 10 μm using a cryostat system on un-coated glass slides prior to DESI-MSI measurement. 

### 4.5. DESI-MSI Analysis

Data were acquired from WT and SAMP8 mice brain sections in positive ionization mode using Xevo G2-XS quadrupole time of flight (Q-TOF) mass spectrometer (Waters, Milford, MA, USA) coupled to a DESI source. Using a solution of sodium formate (500 µM) in 90% 2-propanol (10: 90; water: 2-propanol, *v/v*), mass spectra were calibrated prior to the measurement. As spray solvent, 98% methanol (98:2; methanol: water, *v/v*) was sprayed at 2 µL/min. Ions from the sagittal brain sections were obtained over *m/z* range of 500–1000 (Dalton) Da. Following parameters were used for the optimization of DESI to get best signal intensity on tissue prior to acquire data: capillary voltage, 4.0 kV; cone voltage, 40 eV; capillary temperature, 130 °C; nebulizing gas (nitrogen gas) pressure, 4.0 bar; spatial resolution, 100 μm; incidence angle of sprayer, 75 degree; inlet to sprayer distance, about 10 mm; sample to sprayer distance, about 1 mm; scan speed, 200 μm/sec and mass window of 0.02 Da. For better mass accuracy, lock mass correction was performed using *m/z* 798.5410; exact *m/z* of PC (34:1), the most abundant PC found in brain [48].

### 4.6. LC-ESI-MS/MS Analysis

The lipids from SAMP8 mice brain were extracted using Bligh and Dyer extraction method, with slight modifications [49]. To 2 gm of SAMP8 mouse brain, 2 mL of methanol and 1 mL of chloroform were added, mixed well, and stored at −20 °C for overnight. After that, 1 mL of chloroform and 0.8 mL of 0.28 M acetate were added, and mixed well. Additional 1 mL of 0.28 M acetate was added and mix well, then centrifuged the mixture at 4 °C with 1500 rpm for 10 min. After that, lipid containing lower phase was transferred into new glass tubes and evaporated under vacuum condition using freeze dyer at 0 °C. Finally, dried lipids were dissolved into 2 mL of methanol, then subjected to LC-ESI-MS/MS analysis using 40 eV collision energy in positive ionization mode. The LC–ESI-MS/MS system was consisted of an Acquity UPLC H-Class system (Waters, Milford, MA, USA) coupled to a Synapt G2 Q-TOF mass spectrometer (high definition mass spectrometry, Waters Corp., Manchester, UK). Chromatographic separation was performed by injecting 5 μL sample onto a L-column2 ODS C18 column (2.1 × 150 mm, 2.0 μm particle size, Chemicals Evaluation and Research Institute, Japan). Mobile phase A was consisted of ultrapure water containing 0.2% formic acid and 10 mM ammonium acetate, and mobile phase B was consisted of acetonitrile containing 0.2% formic acid and 10 mM ammonium acetate. The following gradient was used for the elution of PCs (time: %A/%B): 0–50 min: 80/20; 50–60 min: 0/100; 60–60.1 min: 80/20 and 60.1–70 min: 80/20. The flow rate was 0.3 mL/min for elution. The column temperature and autosampler temperature were kept at 50 °C and 10 °C, respectively. The LC eluate was delivered into Synapt G2 Q-TOF mass spectrometer equipped with an electrospray ionization source. Glass apparatus were used in all steps to avoid contamination.

### 4.7. DESI-MSI and LC-ESI-MS/MS Data Analysis

For the acquisition and processing of DESI-MSI and LC-ESI-MS/MS data, MassLynx (Waters, Milford, MA, USA; version 4.1) software was used. For the analysis of ion images, IMAGE REVEAL (Shimadzu, Kyoto, Japan; version 1.0.1.8345) and HDImaging (Waters, Milford, MA, USA; version 1.4) software were used. LC-ESI-MS/MS data analysis was performed by MassLynx software. MS Excel (version 2019) and SPSS (version 16) software were used for all statistical analyses. All data are expressed as mean ± standard error of the mean (SEM), and considered statistically significant at *p* < 0.05.

## 5. Conclusions

In summary, our study demonstrated that GNO or DHA supplementation can restore decreased distribution levels of DHA-PCs in the brain of SAMP8 mice; a mouse model of dementia. After GNO or DHA supplementation, increased accumulation of DHA-PCs in the hippocampus—as well as in the cerebellum, cerebral cortex, caudate putamen, and ventral striatum—were also noted in this study. DHA-PCs play crucial role in brain development and neuronal function. Recently, we have also reported that GNO or DHA supplementation could ameliorate decreased distribution levels of brain DHA and memory function in the same model mice of dementia [10]. Therefore, this study and our previous study explore the possible potentiality of GNO or DHA for the prevention and treatment of dementia. Moreover, all these findings also revealed the possibility of GNO as a source of DHA alternative to fish oil in the future.

## Figures and Tables

**Table 1:** List of precursor ions and molecular assignment based on their mass accuracy, LC-ESI-MS/MS product ions and previous reports

Observed *m/z*	Theoretical *m/z*	Error (ppm)	MS/MS Product Ions (Observed)	MS/MS Product Ions (Reported)	Assigned Molecules
806.5654	806.5694	4.96	86.1, 104.1, 125.0, 184.1, 478.3, 496.3, 550.3, 568.3, 623.5, 747.5	86.1, 104.1, 125.0, 184.1, 478.3, 496.3, 550.3, 568.3, 623.5, 747.5 [23,24,25]	[PC(16:0_22:6)+H]^+^
834.5917	834.6007	10.78	86.1, 104.1, 125.0, 184.1, 506.4, 524.4, 550.3, 568.3, 651.5	86.1, 104.1, 125.0, 184.1, 506.4, 524.4, 550.3, 568.3, 651.5, 775.5 [23,24,25]	[PC(18:0_22:6)+H]^+^
844.5244	844.5256	1.42	86.1, 104.1, 125.0, 184.1, 478.3, 550.3, 623.5, 785.3, 806.6	86.1, 104.1, 125.0, 184.1, 478.3, 496.3, 550.3, 568.34, 623.5, 747.5 785.3, 806.6 [23,24,25]	[PC(16:0_22:6)+K]^+^
872.5559	872.5566	0.80	86.1, 104.1, 125.0, 184.1, 506.4, 550.3, 651.5, 813.4, 834.6	86.1, 104.1, 125.0, 184.1, 506.4, 524.4, 550.3, 568.3, 651.5, 775.5, 813.4, 834.6 [23,24,25]	[PC(18:0_22:6)+K]^+^

## Supplementary Materials

The following are available online at https://www.mdpi.com/2218-1989/10/4/153/s1, Figure S1: Representative mass spectra of DESI-MSI acquired in positive ionization mode from the sagittal brain sections of 14-week-old WT and SAMP8 mice. Figure S2: Average intensity of the distribution of DHA-PCs in the brain of 14-week-old WT and SAMP8 mice (data from three independent experiments),Table S1: Fold decreases in the average intensity of the distribution of DHA-PCs in different brain regions of 14-week-old SAMP8 mice compared to WT mice, Figure S3: Average intensity of the distribution of DHA-PCs in the brain of 28-week-old CO, GNO, and DHA treated SAMP8 mice (data from five independent experiments), Table S2: Fold increases in the average intensity of the distribution of DHA-PCs in different brain regions of GNO or DHA treated SAMP8 mice (28 weeks old) compared to CO treated SAMP8 mice.
