# Supplementary material for: Green Nut Oil or DHA Supplementation Restored Decreased Distribution Levels of DHA Containing Phosphatidylcholines in the Brain of a Mouse Model of Dementia
Source: Metabolites. 2020 Apr 16;10(4):153. doi: 10.3390/metabo10040153 (PMC7240946; doi:10.3390/metabo10040153)
Supplement: Supplementary file 1 [file metabolites-10-00153-s001.zip › Supplementary_Materials.pdf]

Supplementary Materials

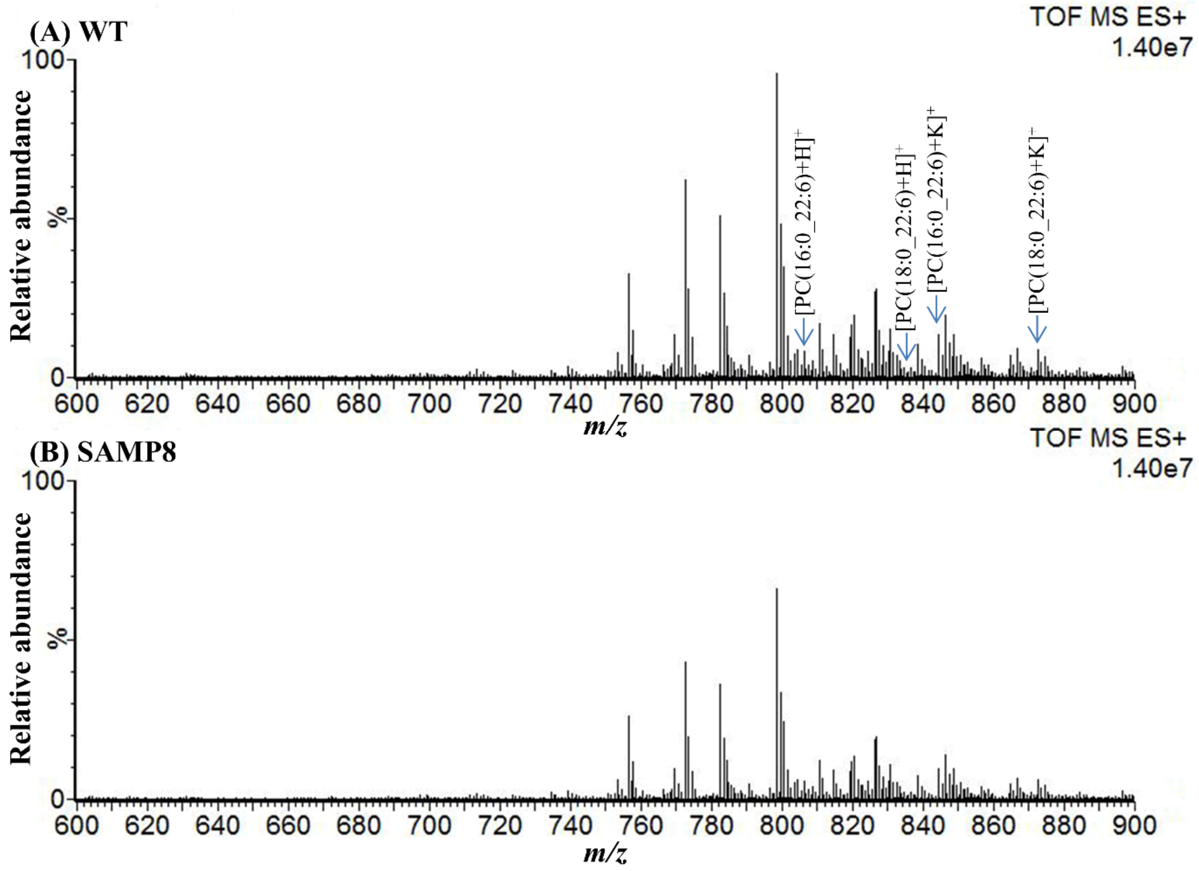

Figure S1: Representative mass spectra of DESI-MSI acquired in positive ionization mode from the sagittal brain sections of 14-week-old WT and SAMP8 mice. WT: wild type mice; SAMP8: senescence accelerated mouse prone 8; PC: phosphatidylcholine and *m*/z: mass to charge ratio.

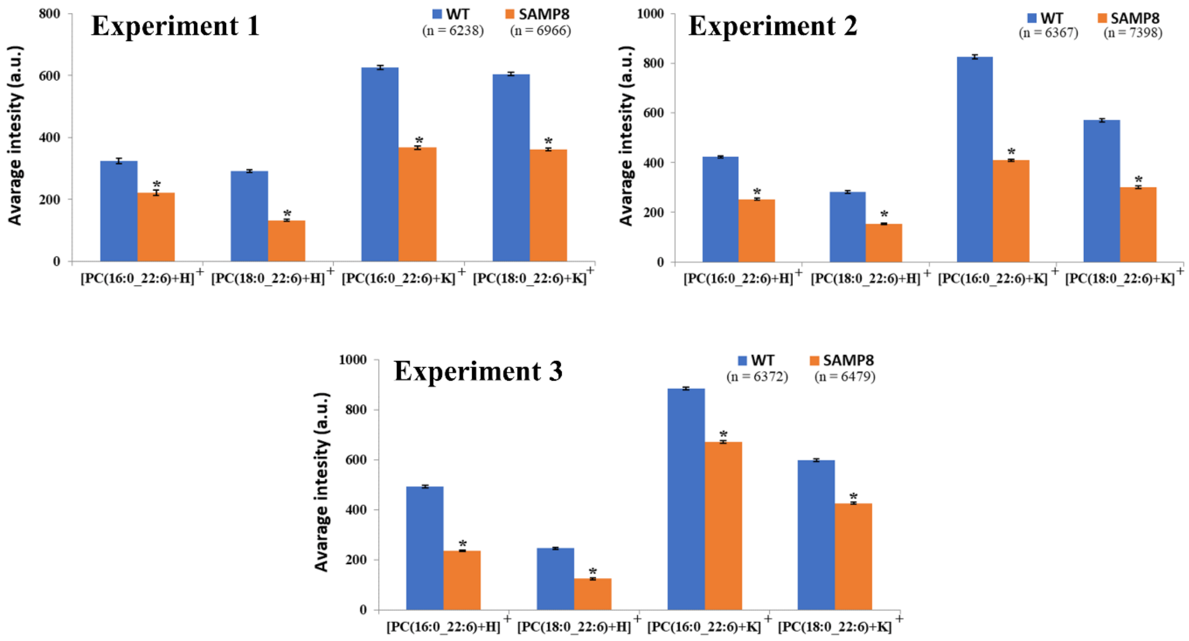

Figure S2: Average intensity of the distribution of DHA-PCs in the brain of 14-week-old WT and SAMP8 mice (data from three independent experiments). All values are expressed as mean ± SEM. Here `a.u.` indicates arbitrary unit, `n` indicates number of pixels, and `*` indicates $P < 0.05$ compared to WT mice (two tailed t-test).

Table S1: Fold decreases in the average intensity of the distribution of DHA-PCs in different brain regions of 14-week-old SAMP8 mice compared to WT mice.

	**DHA containing phosphatidylcholines**			
**Brain regions**	**$[PC(16:0\_22:6)+H]^+$**	**$[PC(18:0\_22:6)+H]^+$**	**$[PC(16:0\_22:6)+K]^+$**	**$[PC(18:0\_22:6)+K]^+$**
Cerebellum (Cb)	1.58 ± 0.26	2.49 ± 0.24	1.8 ± 0.16	1.99 ± 0.22
Cerebral Cortex (Cx)	2.3 ± 0.23	1.9 ± 0.23	2.29 ± 0.34	1.45 ± 0.10
Hippocampus (Hip)	2.51 ± 0.22	2.08 ± 0.09	1.67 ± 0.12	1.78 ± 0.13
Caudate putamen (Cp)	2.37 ± 0.01	2.14 ± 0.18	1.94 ± 0.19	2.08 ± 0.17
Ventral striatum (Vs)	1.99 ± 0.15	2.00 ± 0.12	1.79 ± 0.15	1.48 ± 0.10

All values are expressed as mean ± SEM (n = 3; mice number)

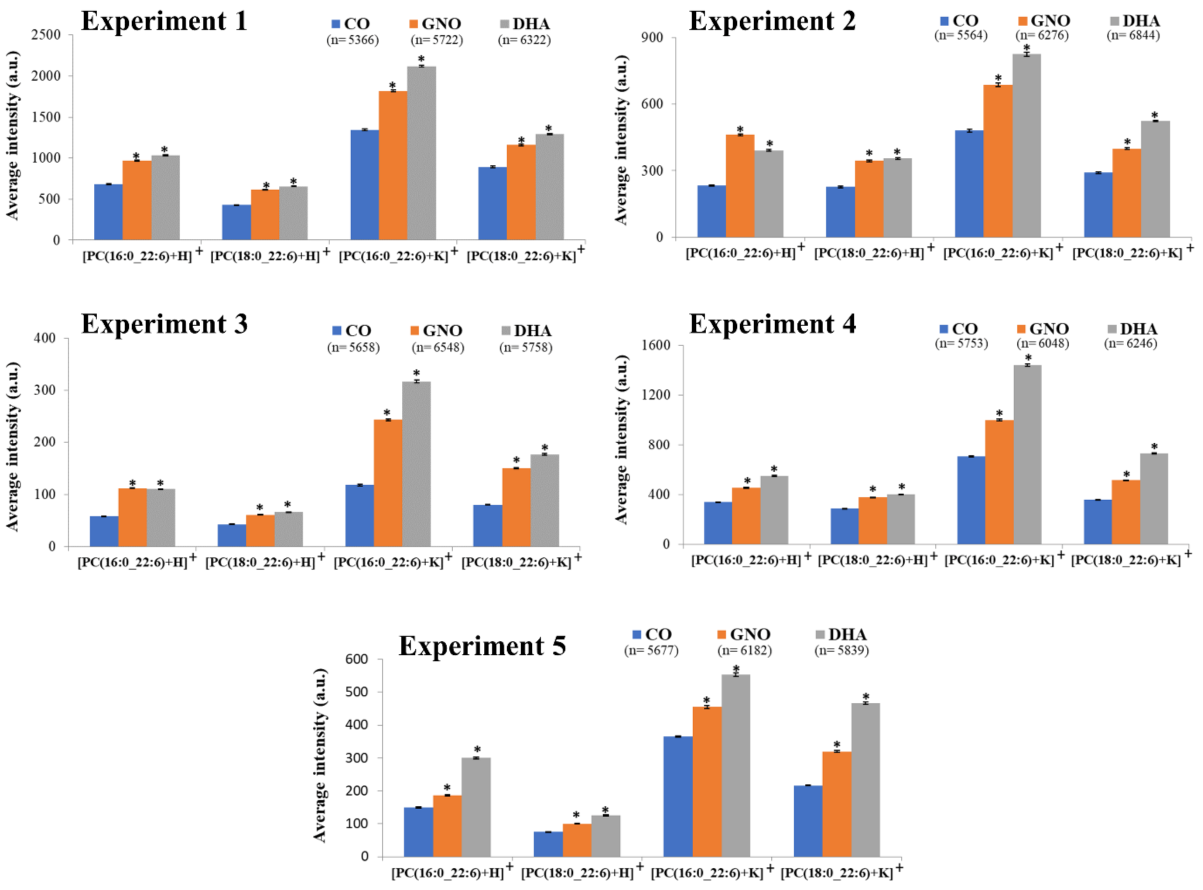

Figure S3: Average intensity of the distribution of DHA-PCs in the brain of 28-week-old CO, GNO, and DHA treated SAMP8 mice (data from five independent experiments). All values are expressed as mean ± SEM. CO: corn oil fed SAMP8 mice; GNO: green nut oil fed SAMP8 mice and DHA: DHA fed SAMP8 mice. Here \`a.u.\` indicates arbitrary unit, \`n\` indicates number of pixels, and \`*\` indicates $P < 0.05$ compared to CO-fed SAMP8 mice (one-way ANOVA with Tukey's multiple comparisons).

Table S2: Fold increases in the average intensity of the distribution of DHA-PCs in different brain regions of GNO or DHA treated SAMP8 mice (28 weeks old) compared to CO treated SAMP8 mice.

**Brain regions**	**DHA containing phosphatidylcholines**			
**GNO treated SAMP8 mice**	**[PC(16:0_22:6)+H]$^+$**	**[PC(18:0_22:6)+H]$^+$**	**[PC(16:0_22:6)+K]$^+$**	**[PC(18:0_22:6)+K]$^+$**
Cerebellum (Cb)	1.33 ± 0.09	1.46 ± 0.0.08	1.56 ± 0.14	1.50 ± 0.15
Cerebral Cortex (Cx)	1.56 ± 0.19	1.42 ± 0.04	1.79 ± 0.16	1.61 ± 0.07
Hippocampus (Hip)	1.71 ± 0.33	1.36 ± 0.10	2.08 ± 0.66	1.92 ± 0.41
Caudate putamen (Cp)	1.99 ± 0.35	1.63 ± 0.08	2.10 ± 0.48	1.98 ± 0.37
Ventral striatum (Vs)	1.97 ± 0.27	1.59 ± 0.08	1.79 ± 0.24	1.81 ± 0.22
**GNO treated SAMP8 mice**				
Cerebellum (Cb)	1.43 ± 0.13	1.59 ± 0.14	2.02 ± 0.18	1.93 ± 0.17
Cerebral Cortex (Cx)	1.68 ± 0.21	1.62 ± 0.08	2.50 ± 0.29	1.93 ± 0.16
Hippocampus (Hip)	1.89 ± 0.23	1.90 ± 0.38	2.55 ± 0.57	2.11 ± 0.36
Caudate putamen (Cp)	2.25 ± 0.40	1.75 ± 0.17	2.24 ± 0.42	1.99 ± 0.38
Ventral striatum (Vs)	2.38 ± 0.37	1.89 ± 0.03	2.21 ± 0.32	2.05 ± 0.31

All values are expressed as mean ± SEM (n = 5; mice number)
