# Supplementary figures and images for: Green Nut Oil or DHA Supplementation Restored Decreased Distribution Levels of DHA Containing Phosphatidylcholines in the Brain of a Mouse Model of Dementia
Source: Metabolites. 2020 Apr 16;10(4):153. doi: 10.3390/metabo10040153 (PMC7240946; doi:10.3390/metabo10040153)

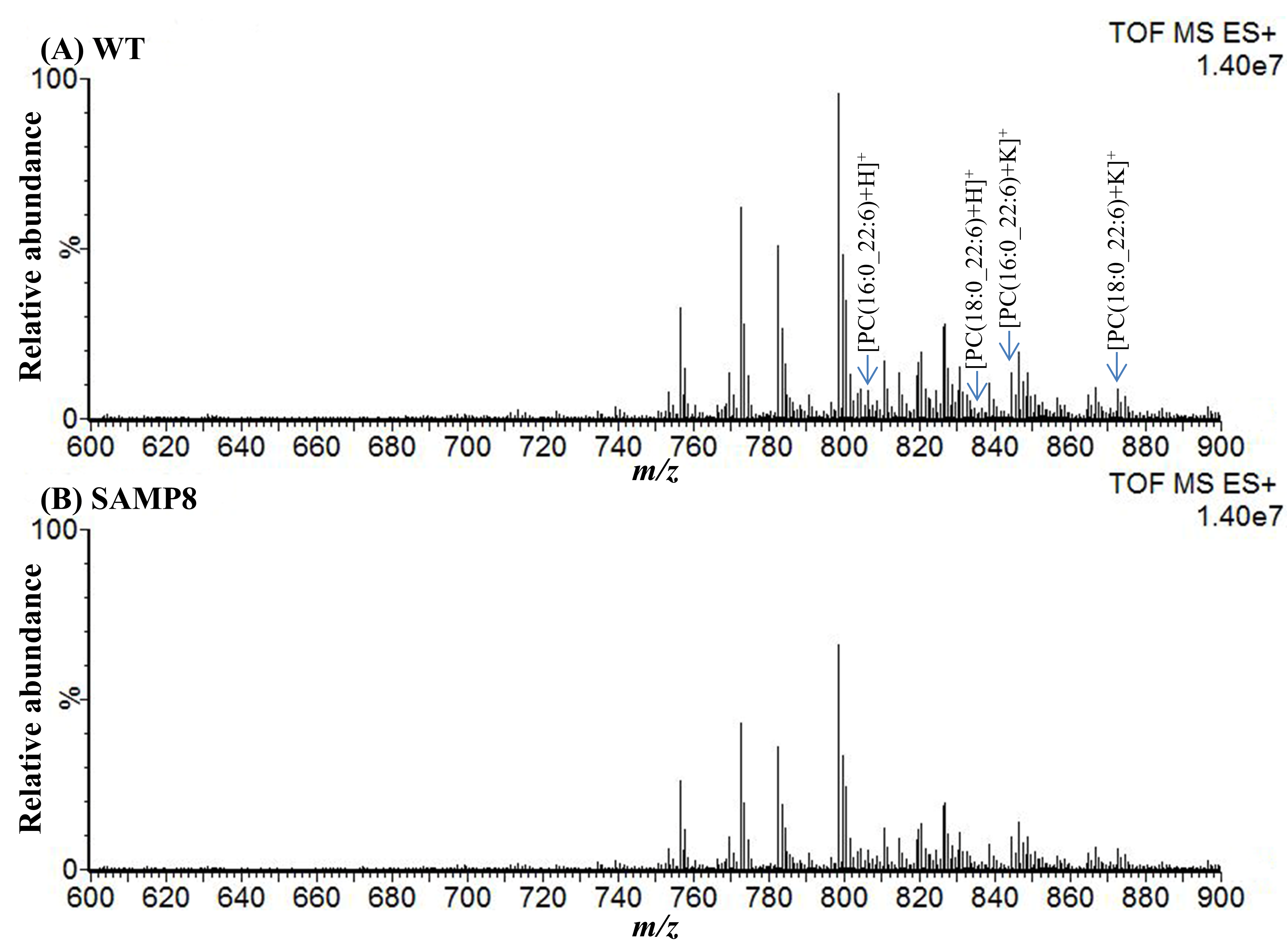

Supplement: Supplementary file 1 [file metabolites-10-00153-s001.zip › Figure S1.tif]

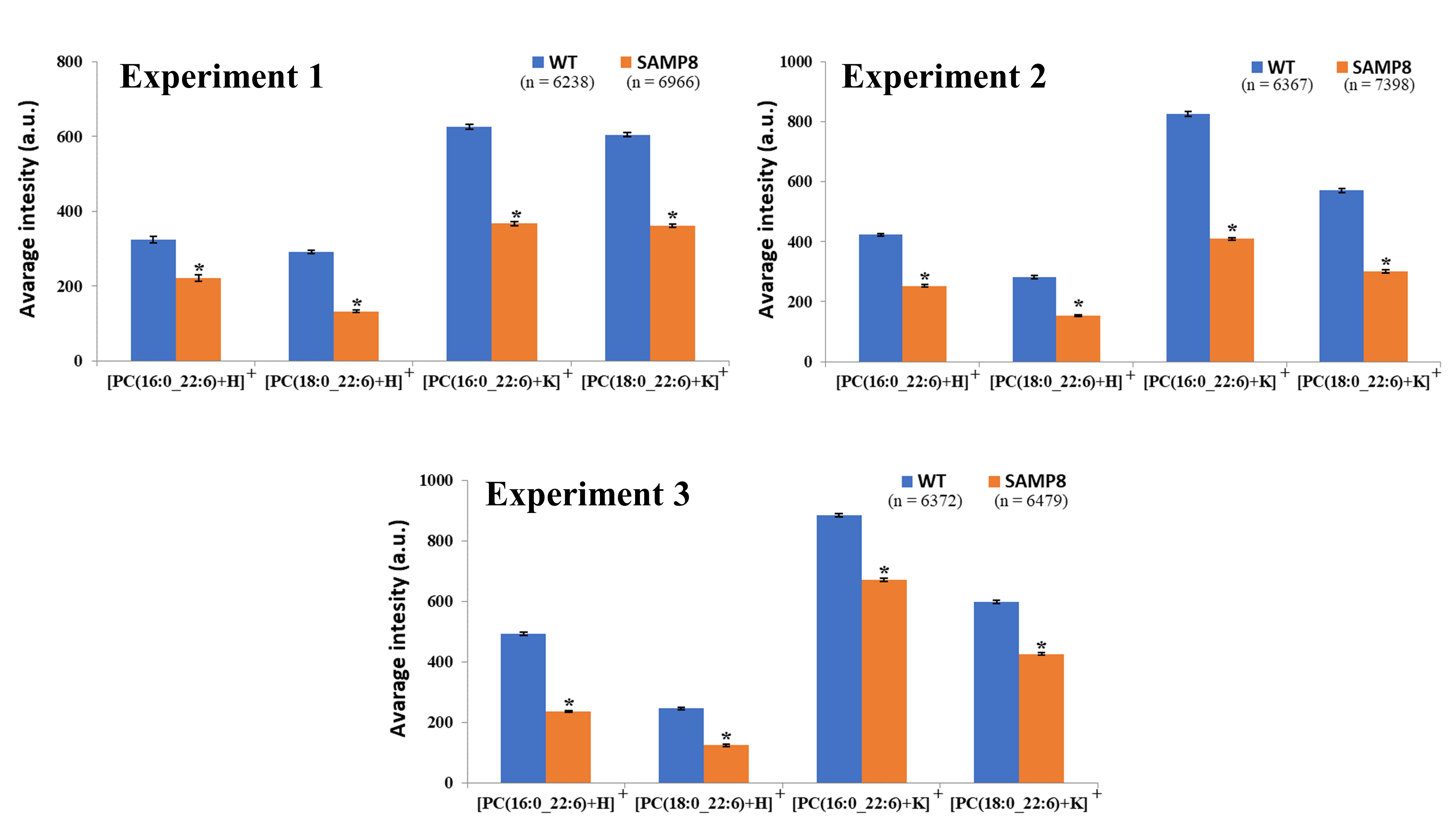

Supplement: Supplementary file 1 [file metabolites-10-00153-s001.zip › Figure S2.tif]

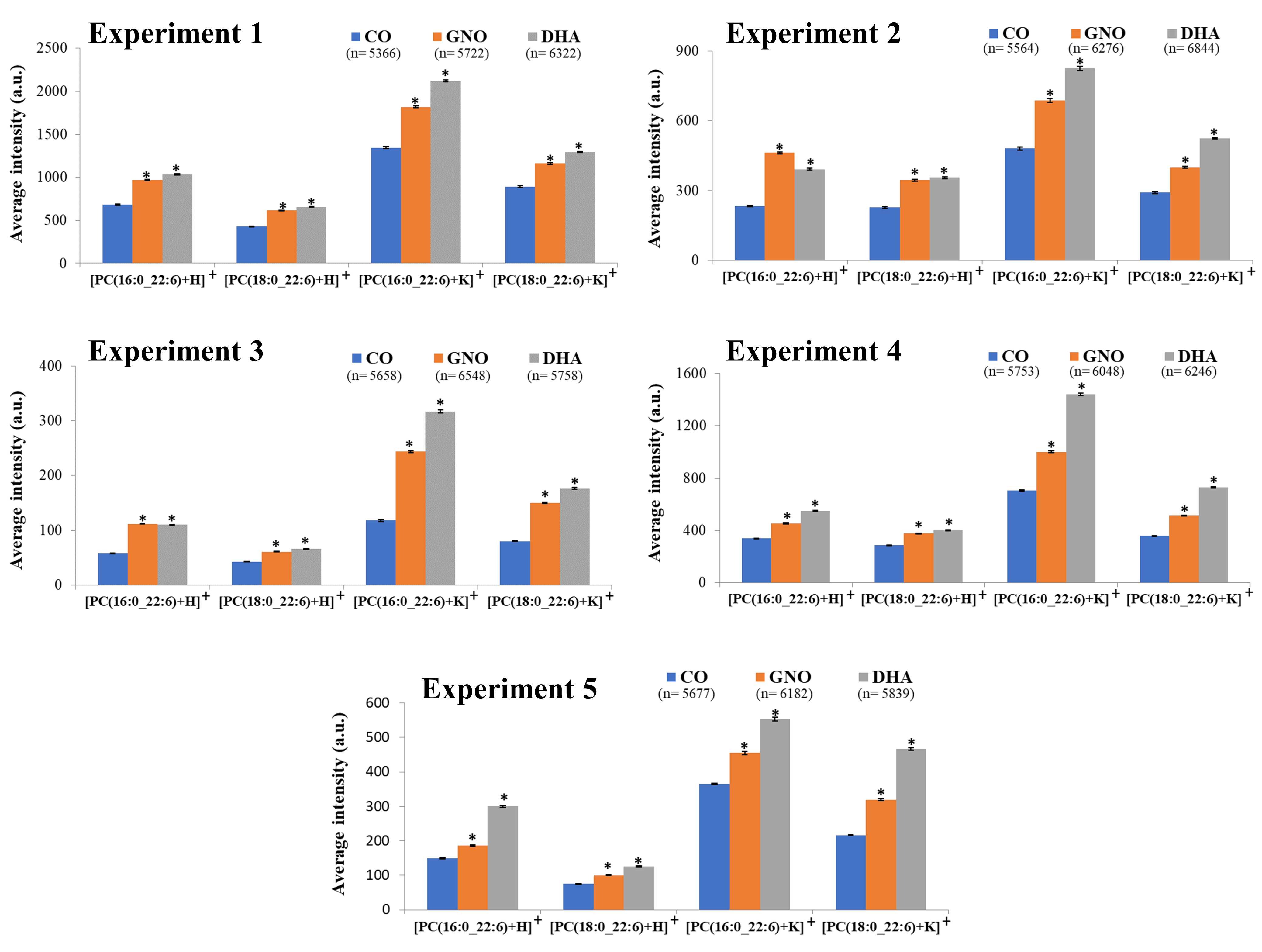

Supplement: Supplementary file 1 [file metabolites-10-00153-s001.zip › Figure S3.tif]
